# Supplementary material for: Laser Synthesis of Platinum Single-Atom Catalysts for Hydrogen Evolution Reaction
Source: Nanomaterials (Basel). 2025 Jan 6;15(1):78. doi: 10.3390/nano15010078 (PMC11723019; doi:10.3390/nano15010078)
Supplement: Supplementary file 1 [file nanomaterials-15-00078-s001.zip › nanomaterials-3337681-supplementary.pdf]

Supplementary information for

Laser Synthesis of Platinum Single-Atom Catalysts for Hydrogen Evolution Reaction

Hengyi Guo[1], Lingtao Wang[1], Xuzhao Liu[1,2], Paul Mativenga[3], Zhu Liu[4,*], and Andrew G. Thomas[1,2,5,*].

[1] Department of Materials, School of Natural Science, The University of Manchester, Oxford Road, Manchester, M13 9PL, UK.

[2] The Photon Science Institute, The University of Manchester, Oxford Road, Manchester, M13 9PL, UK.

[3] Department of Mechanical and Aerospace Engineering, The University of Manchester, Oxford Road, Manchester, M13 9PL, UK.

[4] Ningbo *Institute of Materials Engineering and Technology, Chinese Academy of Sciences, China.*

[5] *The Henry Royce Institute,* The University of Manchester, Oxford Road, Manchester, M13 9PL, UK.

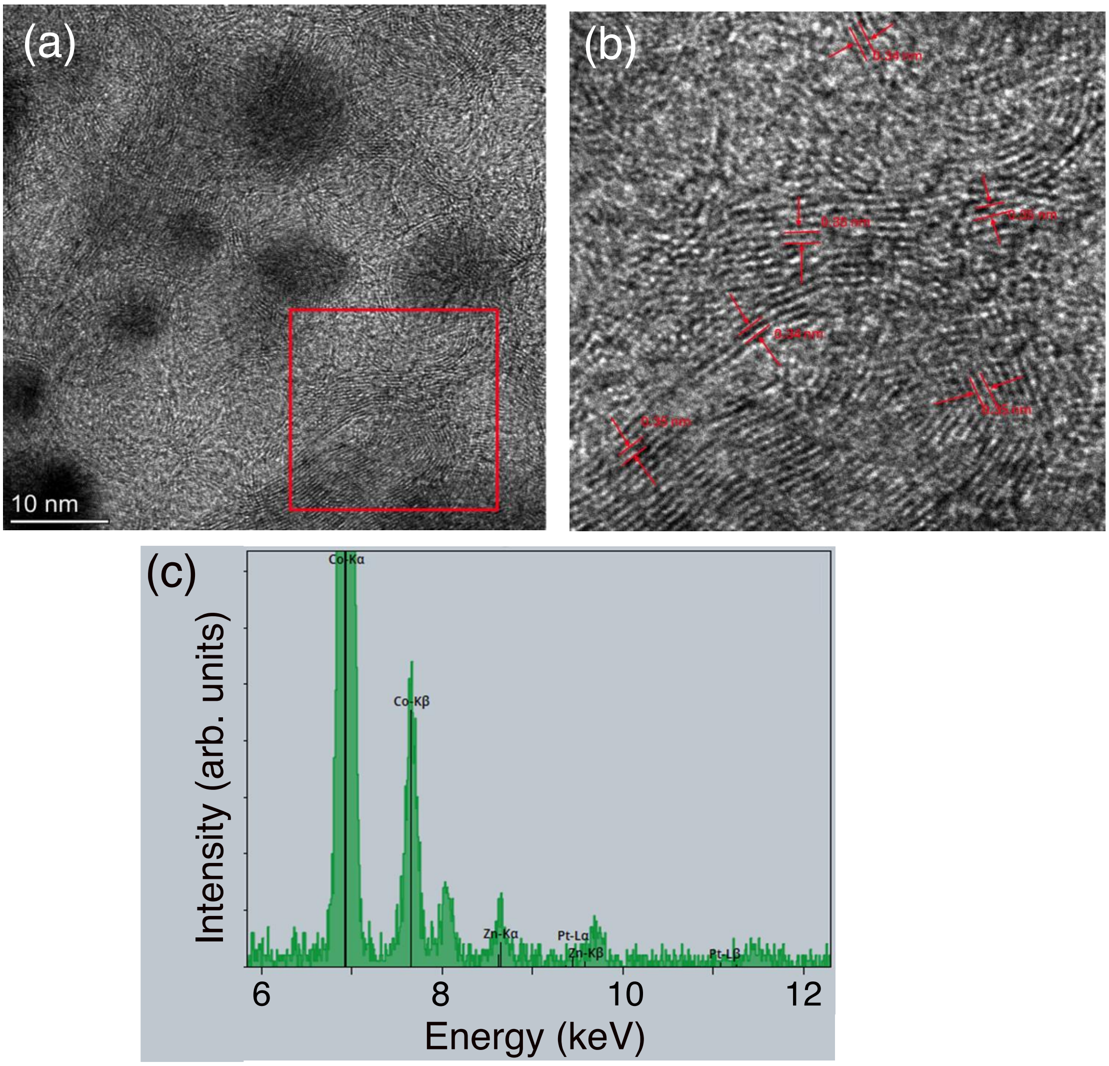

Figure S1. (a) STEM image of the ZIF-8@ZIF-67 after first IR laser treatment (Figure 2(d) in main manuscript) (b) zoomed in image of the region marked by the red square in Figure S1(a), showing the C-C interplanar spacings more clearly, (c) average EDS spectrum of ZIF-8@ZIF-67 after first IR laser treatment (See Figure 3 main manuscript).

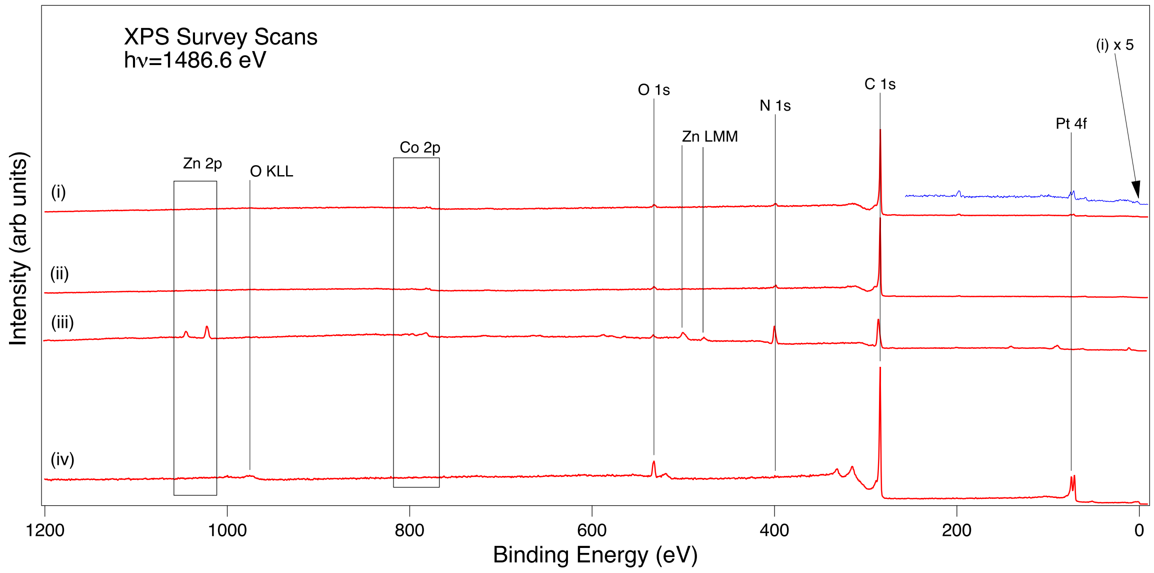

Figure S2. XPS survey spectra recorded from the LIA MOFs. (i) is the ZIF8@ZIF67 MOF sample which has been treated with the 1064 nm laser, Pt precursor and UV marker laser, ii) is the 1067 nm irradiated MOF, iii) is the as-prepared MOF and iv) the commercial Pt-C catalyst sample. The blue line labelled (i) x 5 is a zoomed in portion of spectrum (i) showing the Pt signal.

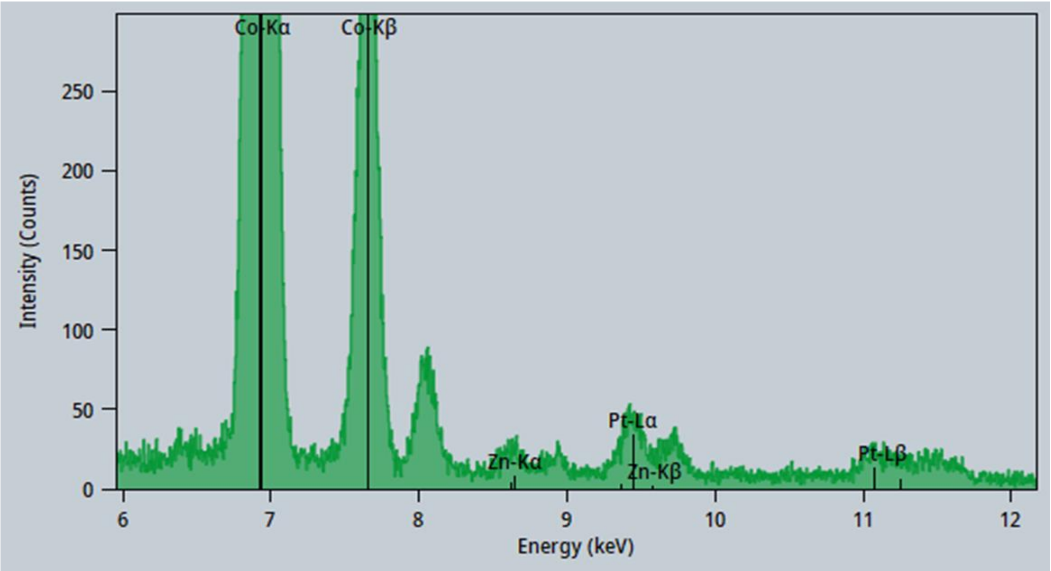

Figure S3. Average EDS spectrum of Pt single atom decorated sample (see Figure 7 main manuscript).

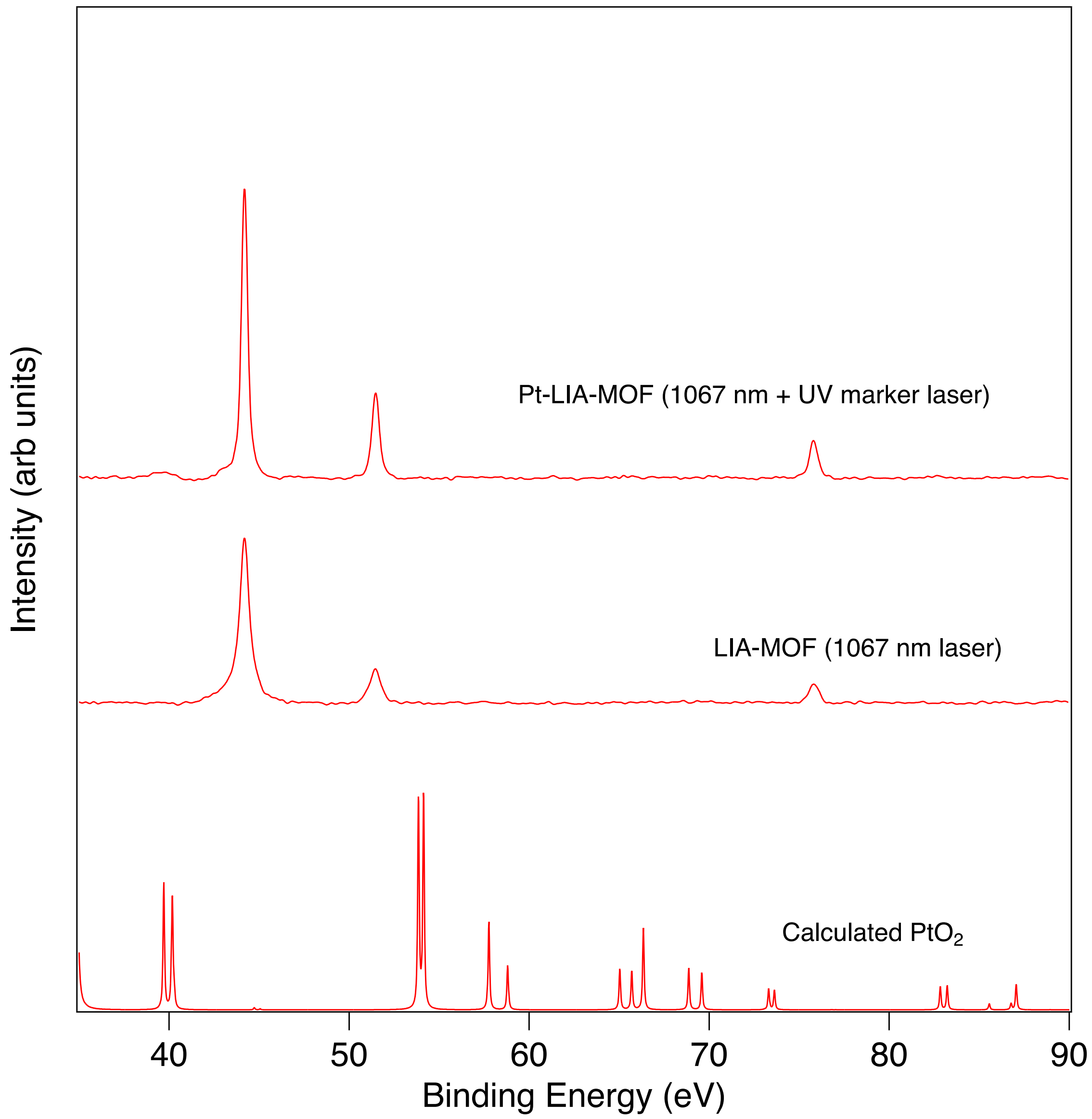

Figure S4. XRD patterns recorded from the chloroplatinic acid infused irradiated MOF, following further irradiation with the UV laser (centre wavelength = 355 nm) (top), the IR irradiated MOF (centre) and the calculated XRD pattern for PtO_2 (bottom). It is clear that no peaks from PtO_2 can be observed in the experimental spectra.

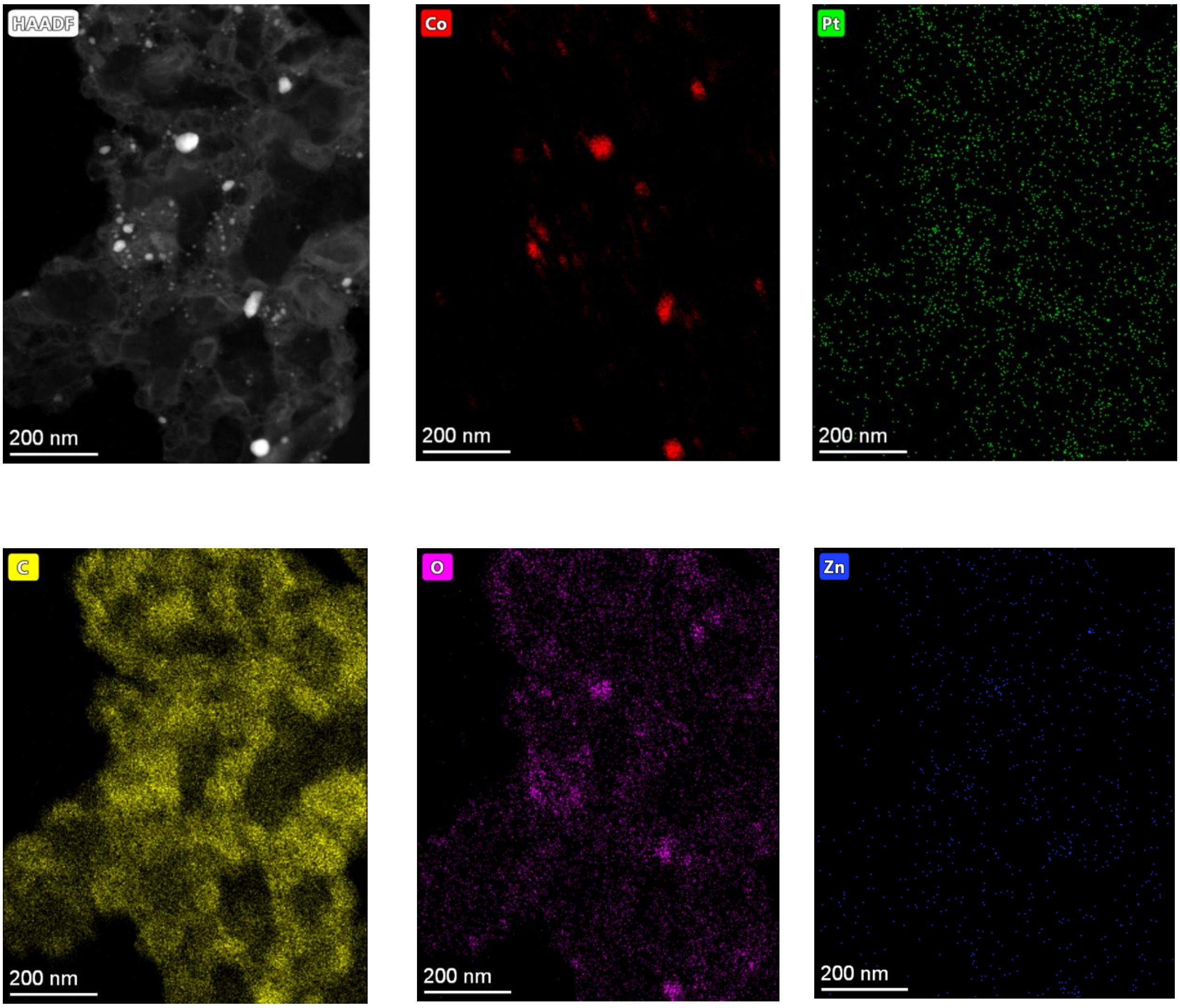

Figure S5. STEM HAADF and EDS maps of the Pt5-LIA-ZIF67@ZIF8 following an acid wash with 0.5 M H_2SO_4.

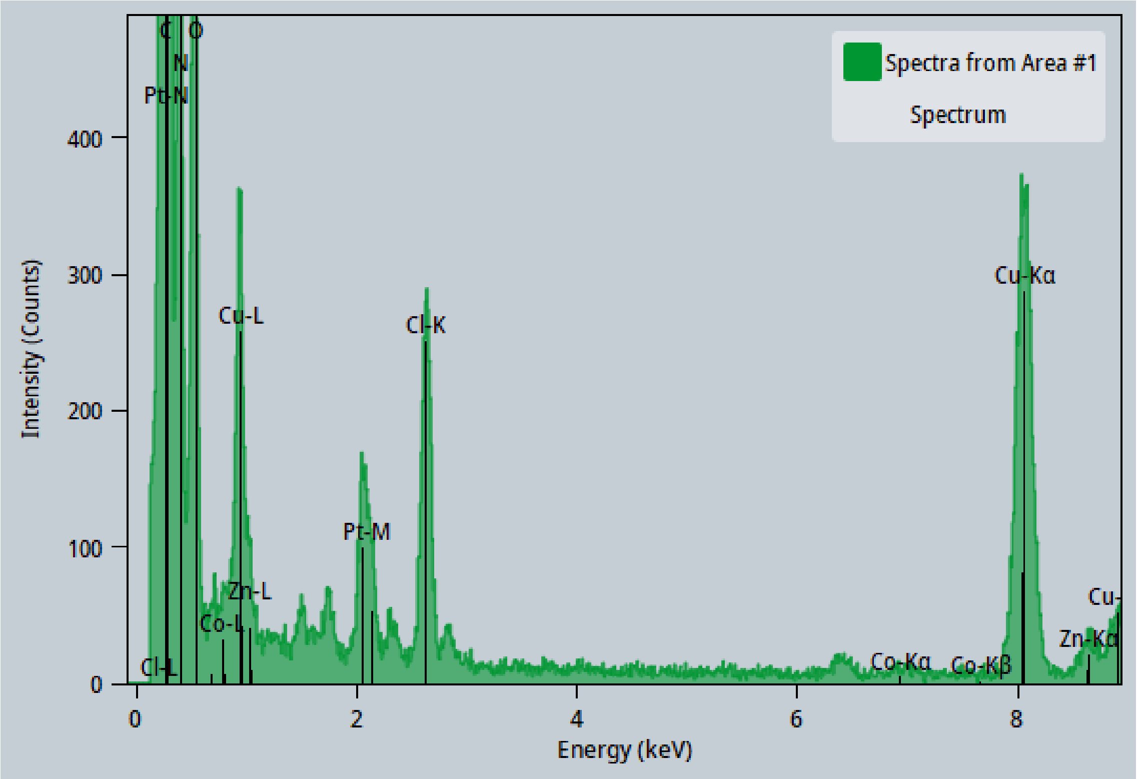

Figure S6. STEM EDS spectrum of the Pt5-LIA-ZIF67@ZIF8 following an acid wash with 0.5 M H_2SO_4. For the sample shown in Figure 10 of the main manuscript.
